# Relatives’ perceived quality of palliative care: comparisons between care settings in which patients die

**DOI:** 10.1186/s12904-017-0224-x

**Published:** 2017-08-16

**Authors:** Dolf de Boer, Jolien M. Hofstede, Anke J. E. de Veer, Natasja J. H. Raijmakers, Anneke L. Francke

**Affiliations:** 1 NIVEL, Netherlands Institute of Health Services Research, P.O. Box 1568, 3500 Utrecht, BN Netherlands; 2 Department of Public and Occupational Health, EMGO Institute for Health and Care Research, VU University Medical Center, P.O. Box 7057, 1007 Amsterdam, MB Netherlands; 3 Expertise Center for Palliative Care Amsterdam, VU University Medical Center, Amsterdam, Netherlands

**Keywords:** Palliative care, Quality of health care, Home care service, Nursing home, Hospice, Hospital

## Abstract

**Background:**

Dying in the preferred setting is an indicator of good palliative care quality. Most people prefer to die at home. But does the quality of care as perceived by their relatives vary depending on the care setting that is the place of death?

The aim is to compare (from the relatives perspective) whether there are perceived differences in the quality of palliative care between the settings in which people die.

**Methods:**

Multivariate linear regression analyses have been carried out using an existing dataset containing information collected using the relatives’ version of the Consumer Quality Index (CQ-index) Palliative Care. The dataset includes 1368 relatives of patients with a wide variety of conditions who died in various locations: at home, in hospital, in residential care for the elderly, a hospice, palliative care unit or in another institution (e.g. institutions for people with intellectual disabilities or mental healthcare institutions). The relatives were the first contacts (family members or other people close to the patient) and they received the survey between 6 weeks and 6 months after the bereavement.

**Results:**

Based on the raw data, differences between locations in terms of the perceived quality of care initially appeared inconsistent. The multivariate regression analyses however showed that relatives of people who died at home were generally the most positive about the palliative care that the patient and they themselves received when the patient was dying. The care provided by hospices also received a relatively good rating. In hospitals and in residential settings for care of the elderly, the care was rated less highly by the relatives.

**Conclusions:**

The quality of palliative care as experienced from the relatives’ perspective is highest when the patient dies at home or in a hospice. This is an argument for letting people die at home, if they so wish, as far as possible and feasible.

## Background

People stay in various different settings at the end of their lives: at home, in a hospital, in residential care for the elderly or at specialised palliative care facilities such as hospices or palliative care units. Dying in the preferred setting is an indicator of good palliative care quality [1]. Most people would prefer to die at home [2]. There is however a discrepancy between the desired and actual places of death. An international comparative study of death certificates in 14 countries has shown that 13% to 53% of people who die from diseases that are known to be associated with palliative care needs relatively often (cancer, organ failure, COPD, neurological diseases, HIV/AIDS) die at home. Between 25% and 85% die in hospital, 1% to 35% in residential care for the elderly and 4% to 13% in a specific palliative care facility [3]. In the Netherlands, patients die at home relatively often, but in this country too there is a discrepancy between the desired and actual places of death: over a third of Dutch people who die from diseases that are known often to be associated with palliative care die at home [3], which is clearly lower than the 68% who indicated in the study by Koekoek that they would like to die at home [4]. Over a third die in residential care for the elderly and a quarter (25%) die in hospital [3].

An interesting issue is whether the perceived quality of care is associated with the place of death. Earlier research has shown that - according to the relatives - people with cancer who remained at home at the end of their lives perceived a lower burden from their symptoms and the end of their life was better than people with cancer who died elswhere [5]. Relatives of people with dementia who stayed at home were more satisfied with the care [6] and relatives of people with chronic conditions who received care at home indicated that they had fewer care needs that were not met [7]. The care for people who die in hospital was rated less highly (in comparison with the care at home or in a nursing home) by the relatives of people with dementia [6]. In addition, it is known that relatives rate the care received by cancer patients who die in a hospice more highly than the care received by people who die in hospital [8].

Existing research therefore shows that patients prefer to die at home and that relatives are more positive about the care at home or in a hospice than in hospitals. Limitations of the existing studies into the quality of care around the time of death are however that only a single specific group of patients was examined (e.g. cancer or dementia [5, 6, 8]), that they were carried more than a decade ago [7], and/or that not all care settings that are relevant for palliative care were included [7, 8]. In this article, we are therefore reporting a study based on questionnaire data from relatives of people with a wide range of diseases (such as cancer, dementia and various cardiovascular problems and lung disease) who died in various care settings.

The study questions are as follows:

- Are there differences in the perceived quality of care from the relatives’ perspective between the settings where people die? And if so, in which aspects of the quality of care can those differences be found?

On the basis of the studies described above, we expect that relatives will perceive the quality of care to be better when the patient dies at home. This is particularly the case for aspects of care in which the bond between the caregiver and the care recipient plays a major role. In the Netherlands, people often have long-term patient/doctor relationships with the same general practitioner. They also sometimes receive care at the end of their lives for weeks or months from nursing staff or carers from the home care services. In addition, we expect that relatives of people who die in specialised palliative care institutions (a hospice or palliative care unit) will have relatively good experiences with the care provided. We expect that this will apply in particular to aspects such as expertise and the provision of information, because of the palliative care skills and specialisation that such settings have.

## Methods

### Description of the dataset used

We used an existing dataset for this article that is derived from questionnaires in the evaluation of the Dutch National Quality Improvement Programme for Palliative Care [9]. We only used data from the baseline measurement (i.e. before the quality improvement measures were started), which was available in April 2016. The data analysed was collected using the relatives’ version of the CQ-index Palliative Care [10]. This is a reliable, validated questionnaire that is suitable for measuring the quality of care as experienced in various care settings for people with a wide range of diseases. The healthcare professionals participating in the palliative care improvement programme collected contact details of the bereaved relatives who met the inclusion criteria (e.g. via registration systems within their care organisations or from medical records). The data was collected for various care settings using postal questionnaires: at home, in hospital, in residential care for the elderly (nursing homes or care homes), in hospices, palliative care units or in other settings such as institutions for people with intellectual disabilities or mental healthcare institutions. The relatives in question were the first contacts (family members or other people close to the patients) who were involved in the care of a patient who died after a period of disease and where the death had occurred between 6 weeks and 6 months earlier. The overall response of bereaved relatives in the National Quality Improvement Programme is approximately 50%.

The relatives’ version of the CQ-index Palliative Care measures both the perceptions of care for the patient themselves in the final week before death and the support received by those close to them in the final week before death and after the patient’s death [10]. The questionnaire consists of thirty pre-structured questions with four response categories, such as “Did the care providers have enough time for you?” (none of them, some, most of them, all). In addition, five questions have two possible responses, such as “Were the possibilities of follow-up care for you after your loved one died pointed out to you?” (yes/no). Three questions also asked about the general rating of the care for both the patient and the relative during the final week before death and for the support after death, on a scale from 0 (extremely poor care) to 10 (excellent care). The questionnaire also covers background characteristics of both the deceased patient and the relative who completed the questionnaire.

### Data analysis

The questions in the relatives’ version of the CQ-index Palliative Care yielded six scales [10], which were analysed as dependent variables. Two of the scales address the quality of care that was provided to the patient (care for the psychosocial/spiritual well-being of the patient, expertise) and four of the scales address the support provided for the relatives themselves (care for the psychosocial/spiritual well-being of the relatives, attitude to them, respect for autonomy, and information provided to the relative during the final week before death). An average score (from 1 to 4) was calculated for each scale, with a higher score representing a more positive experience for the subject concerned. In addition to the scales, the rating score was analysed as a dependent variable.

Because there are indications that not only the place of death but also characteristics of the patient (such as the disease [11]) and background characteristics of the respondents (such as age, level of education and state of health [12]) can affect the perceived quality of care, these characteristics were also included as independent variables and always estimated in four multivariate linear regression models:

- Model 1: only the background characteristics of the patients and relatives
- Model 2: background characteristics and place of death
- Model 3: background characteristics and disease characteristics
- Model 4: background characteristics, place of death and disease characteristics

The explained variances have been reported for all the models. Likelihood ratio tests have been performed to assess whether models 2 and 3 fit the data significantly better than model 1. Finally, model 4 was tested against model 3 to assess whether the place of death contributes significantly to explaining the dependent variable if characteristics of the disease and the background are already in the model. For the sake of conciseness, the coefficients for the place of death have only been reported for the model that fits best (model 4). The analyses were carried out using the statistical software package STATA 13.

## Results

### Characteristics of the patients and their relatives

Of the 1368 relatives in the dataset, two thirds were women (67%). In most cases, they were a spouse/partner (36%) or child (39%) of the deceased patient. The majority had stated that their own health was good or excellent (85%). Nearly three quarters (72%) of the deceased patients were aged 75 or above, approximately half were women (54%) and cancer was the most common disease among the patients (43%), followed by dementia (27%). Of six symptoms listed, the relatives stated that fatigue was the biggest burden for the patient in the final week before death, giving it an average of 8.0 on a scale from 0 to 10. See also Table 1.

**Table 1:** Characteristics of the relatives and deceased patients, *n* = 1368

Relatives		Patients	
	*n (%)*		*N (%)*
Age		Age	
18–54	343 (25)	18–64	154 (12)
55–64	443 (33)	65–74	214 (16)
65–74	340 (25)	≥ 75	961 (72)
≥ 75	231 (17)	Gender	
Gender		Male	630 (46)
Male	447 (33)	Female	734 (54)
Female	911 (67)	Disease^a^	
Relationship to the deceased patient		Cancer	575 (43)
Partner	490 (36)	CVA^b^	187 (14)
Child	668 (39)	COPD^c^	131 (10)
Family, other than	163 (12)	Cardiac failure	195 (14)
partner or child		Dementia	367 (27)
Other (not family)	36 (3)	General deterioration	74 (5)
Educational level		Other	218 (16)
Lowest	114 (9)	Symptom burden (0–10)	*M (SD)*
Lower	526 (40)	Pain	5.59 (3.50)
Secondary	346 (26)	Fatigue	7.95 (2.58)
Higher	325 (25)	Shortness of breath	5.04 (3.71)
Perceived health		Constipation	3.22 (3.41)
Excellent	157 (12)	Sombre mood	4.66 (3.43)
Very good	233 (17)	Anxiety	4.52 (3.55)
Good	766 (57)		
Moderate	184 (14)		
Poor	12 (1)		

^a^More than one disease is possible for each patient

^b^ *CVA* Cerebrovascular accident

^c^ *COPD* Chronic Obstructive Pulmonary Disease

The largest group of patients was those dying in residential care for the elderly (a nursing home or care home) (41%). One third (32%) died at home, 11% in hospital, 9% in a hospice, 6% in a palliative care unit at a nursing home or care home, and 2% in another institution (largely institutions for the intellectually disabled or for mental healthcare).

### The perceived quality of palliative care

The care provided to the patient in the last week of life was rated at 8.5 on a scale from 0 to 10 (raw means; see Table 2). Care in hospices received the best score at 9.1; hospital care scored lowest at 7.8. The care provided for people who died at home was given 8.9. Relatives gave the formal caregivers an average rating of 8.3 for the support they themselves received in the final week before death and 7.8 for the support after the death. The highest scores for these aspects were given to support in other institutions (primarily care for the intellectually disabled and mental healthcare), with the lowest scores for support from hospitals.

**Table 2:** Quality of palliative care for various places of dead

	Whole group *N* = 1368 (100%)	Home *N* = 430 (32%)	Residential care for the elderly *N* = 559 (41%)	Hospital *N* = 148 (11%)	Hospice *N* = 117 (9%)	Palliative care unit^a^ *N* = 76 (6%)	Other institution^b^ *N* = 34 (2%)
	M (SD)	M (SD)	M (SD)	M (SD)	M (SD)	M (SD)	M (SD)
General quality rating (0–10)							
Care provided to the patient in last week of life	8.54 (1.56)	8.87 (1.32)	8.33 (1.58)	7.82 (2.02)	9.11 (1.07)	8.52 (1.58)	8.97 (1.31)
Support by healthcare professionals for relatives before death of the patient	8.26 (1.73)	8.48 (1.64)	8.11 (1.71)	7.50 (2.23)	8.88 (1.13)	8.20 (1.67)	9.03 (1.26)
Support by healthcare professionals for relatives after death of the patient	7.75 (2.23)	7.80 (2.34)	7.76 (2.02)	6.82 (2.80)	8.45 (1.65)	7.67 (2.17)	8.70 (2.02)
CQI^d^ dimensions (1–4)^c^							
Care for the psychosocial/spiritual well-being of the patient	3.65 (0.53)	3.76 (0.42)	3.61 (0.56)	3.46 (0.68)	3.73 (0.47)	3.62 (0.53)	3.75 (0.59)
Care for the relative’s own psychosocial/spiritual well-being	3.42 (0.75)	3.52 (0.68)	3.38 (0.72)	2.96 (1.03)	3.62 (0.66)	3.43 (0.78)	3.62 (0.52)
Attitude to the relatives	3.62 (0.53)	3.76 (0.41)	3.55 (0.54)	3.36 (0.72)	3.76 (0.37)	3.65 (0.45)	3.69 (0.59)
Respect/care for autonomy	3.59 (0.57)	3.76 (0.42)	3.54 (0.58)	3.33 (0.79)	3.63 (0.53)	3.52 (0.57)	3.60 (0.65)
Information for the relative in the last week before death	3.31 (0.62)	3.34 (0.58)	3.31 (0.60)	3.02 (0.73)	3.49 (0.55)	3.31 (0.58)	3.60 (0.64)
Expertise	3.44 (0.56)	3.56 (0.49)	3.35 (0.58)	3.34 (0.65)	3.55 (0.54)	3.39 (0.55)	3.54 (0.55)

^a^Palliative care unit at a nursing home or care home

^b^Other institution, mainly care for the handicapped or mental healthcare

^c^a higher value represents a more positive rating

^d^ *CQI* Consumer Quality Index, a survey for measuring experiences

In addition to the general ratings for the care received, we also looked at specific aspects of care, using scales within the CQ-index Palliative Care. The scales range from 1 to 4, with higher values representing a more positive experience for the subject concerned. The aspect of ‘information provided to relatives in the final week before death’ was rated least positively (3.31, SD 0.62) and the aspect of ‘care for the psychosocial/spiritual well-being of the patient’ highest (3.65, SD 0.53). Once again, the lowest scores on these scales were given by the relatives of people who died in hospital and the highest scores when the patient died at home, in a hospice or in another institution (largely institutions for the intellectually disabled or for mental healthcare).

### Effect of the place of death

In order to examine the effect of the place of death on the quality of care perceived, the explained variance (R^2^) of the various multilinear regression models is shown in Table 3. The explained variance in the model was greater when the characteristics of the disease and the background were added, than if the place of death was added to the background characteristics. The explained variance is greatest for the overall model including background characteristics, place of death and disease characteristics (ranging from 0.064 to 0.119; model 4). The likelihood ratio tests show that both the model containing the background characteristics and the place of death and the model containing the background characteristics and disease characteristics fit the data better than the model containing only the background characteristics (*p* values <0.05). Finally, the model containing the background characteristics, disease characteristics and the place of death was shown to fit the data significantly better than the model containing only the background characteristics and the disease (*p* values <0.05).

**Table 3:** Explained variance of various models (as percentages)

	Adjusted R-squared			
	Model 1	Model 2	Model 3	Model 4
General quality rating (0–10)				
Care provided to the patient in last week of life	0.012	0.056	0.074	0.104
Support by healthcare professionals for relatives before death of the patient	0.014	0.052	0.076	0.100
Support by healthcare professionals for relatives after death of the patient	0.010	0.035	0.046	0.064
CQI^a^ dimensions (1–4)				
Care for the psychosocial/spiritual well-being of the patient	0.019	0.050	0.064	0.087
Care for the relative’s own psychosocial/spiritual well-being	0.024	0.060	0.096	0.119
Attitude to the relatives	0.027	0.075	0.069	0.103
Respect/care for autonomy	0.012	0.061	0.068	0.109
Information for the relative in the last week before death	0.013	0.040	0.042	0.061
Expertise	0.023	0.044	0.068	0.080

Model 1: Background characteristics (age and gender of the patient, age, gender, level of education and perceived health of the relative and their relationship to the patient)

Model 2: Background characteristics + place of death

Model 3: Background characteristics + disease characteristics of the patient (disease and burden of the symptoms)

Model 4: Background characteristics + place of death + disease characteristics

^a^ *CQI* Consumer Quality Index, a survey for measuring experiences

### Differences in the quality of care by place of death

To obtain a picture of the quality of care by the place of death, the overall model containing characteristics of both the background characteristics of patients and relatives and the disease was used. Table 4 shows that the quality of care as experienced by the relatives of patients who died in hospital is rated less positively on all aspects than that perceived by relatives of people who died at home. The same applies when the patient died in residential care for the elderly: this care was rated less positively on 7 of the 9 aspects, compared to the care for patients who died at home. The aspect of ‘respect/care for autonomy’ was rated more highly when people die at home than when they die in any of the other settings. ‘Care for the psychosocial/spiritual well-being of the patient’ was less positively rated by the relatives of people who died in a hospice or the palliative care unit of a nursing home or care home. There were no aspects for which the care given to people who die at home was rated significantly less well than the care for people who die anywhere else.

**Table 4:** Differences in the quality of care or various places of death^a, b, c^

	Home	Residential care for the elderly	Hospital	Hospice	Palliative care unit^d^	Other institution^e^
General quality rating (0–10)						
Care provided to the patient in last week of life	ref	**−0.584**	**−0.921**	0.010	**−0.529**	−0.163
Support by healthcare professionals for relatives before death of the patient	ref	**−0.416**	**−0.892**	0.229	−0.451	0.308
Support by healthcare professionals for relatives after death of the patient	ref	−0.071	**−0.971**	0.582	−0.266	0.396
CQI^f^ dimensions (1–4)						
Care for the psychosocial/spiritual well-being of the patient	ref	**−0.164**	**−0.274**	**−0.167**	**−0.227**	−0.229
Care for the relative’s own psychosocial/spiritual well-being	ref	**−0.188**	**−0.473**	−0.040	−0.139	−0.032
Attitude to the relatives	ref	**−0.187**	**−0.367**	−0.042	−0.136	−0.193
Respect/care for autonomy	ref	**−0.266**	**−0.388**	**−0.176**	**−0.306**	**−0.334**
Information for the relative in the last week before death	ref	−0.071	**−0.294**	0.118	−0.118	0.027
Expertise	ref	**−0.204**	**−0.150**	−0.051	**−0.197**	−0.118

^a^Corrected for age and gender of the patient, age, gender, level of education and perceived health of the relative and their relationship to the patient; characteristics of the patient’s disease (disease and burden of symptoms)

^b^For the sake of brevity, only the coefficients for the place of death have been reported

^c^Items in bold are significantly different from the reference group (= home) (*p* < 0.05)

^d^Palliative care unit at a nursing home or care home

^e^Other institution, mainly care for the handicapped or mental healthcare

^f^ *CQI* Consumer Quality Index, a survey for measuring experiences

## Discussion

When various aspects of palliative care are examined, the quality of palliative care for people who die at home is rated more highly by their relatives than is the case for people who die elsewhere. In particular when the patient dies in hospital or in a residential setting for care for the elderly, the quality of care is perceived as less good by the relatives. Care associated with death in a hospice and in other institutions such as those for the intellectually disabled or mental healthcare services is rated almost as highly as the care for people who die at home.

Perceptions of quality of care at the end of life are affected not only by the place of death, but also by background characteristics and disease characteristics. Our research confirms this: the model containing not only characteristics of the backgrounds and the diseases but also the place of death is better able to explain the differences in the quality of care as experienced than the model containing only the background and disease characteristics. In addition, disease characteristics such as the type of disease can affect the place where someone dies. For instance, people with dementia [13–15] and those who have had a stroke [14] mostly die in a nursing home or care home, and people with cancer die most often (in the Netherlands) in their homes [16]. When people are admitted to hospital shortly before death, this is often because of an acute situation with symptoms such as shortness of breath or digestive or cardiovascular problems [17]. The analyses therefore take account of both the burden of symptoms and the patient’s disease; even after these corrections, the differences in the perceived quality of care between the various care settings remains statistically significant. Nevertheless, the ability of the models to explain the differences is no more than 12%, even with the background and disease characteristics added to the place of death. That means that there are other factors that affect the perceived quality that cannot be accounted for by our data.

The finding that the care provided to people who die at home is rated most positively supports the current Dutch policy of letting people live at home as long as possible [18]. In addition, it is in line with the wishes of many people, who would prefer to die at home [2]. Nevertheless, it is not always either possible or desirable for a patient to die at home. Research by Korte-Verhoef et al. [19] has for instance shown that a large proportion of hospital admissions are avoidable, but at the same time that there can still be situations in which the symptoms are so complex that admission is inevitable. It is therefore important that sufficient attention is paid to good palliative care in settings other than home, and in particular those that are not specialised in palliative care, such as hospitals and residential care for the elderly. A consultation team or specialist palliative team can provide support for this [20, 21]. Our results also demonstrate that this specialist palliative care can offer added value. Experiences with the care in a palliative unit in a nursing home or care home are more positive than those in the regular departments of a nursing home or care home. This applies in particular to the aspects involving care for the relatives. Specialist palliative care is also provided at hospices. The care associated with the patient’s death is also rated relatively highly by the relatives of patients who die in this setting.

The principal reasons for people being admitted to a hospice in the Netherlands are when there is a preference for dying in a hospice and when intensive care and support are needed [22]. This specialised set of knowledge and skills results in good care for both the patient and their loved ones. In addition to the institutions already described, there are also institutions where palliative care is given to specific target groups. In our analysis, the ‘other institutions’ group largely comprises institutions for the intellectually disabled and for mental healthcare. The perceptions of relatives of people who die in these settings are no less positively rated (with the exception of a single aspect) than when people die at home. Because people often live in such institutions for a large proportion of their lives, these institutions can also be seen as a home setting to a certain extent. It is however only a small group within our analyses. To obtain a better picture of the experiences with palliative care in these institutions, further research into these specific target groups is needed.

Our research also looked at the differences in specific aspects of palliative care. In all the care institutions included in this study, the aspect of ‘respect/care for autonomy’ was experienced as being less good than the home setting. This aspect looks at the extent to which the loved ones are involved in decisions made by the caregivers about the care, whether they could determine what their own role was within that care, and whether allowances were made for the individual wishes of the relatives with respect to the care for the patient. One possible explanation for this is that the partners of people who die at home often live in the same house and are therefore closely involved in the care and also tend to take on the role of informal caregiver. Previous research has also shown that it is important for the relatives of people who die at home to be included as part of a good professional care team, and that they have the opportunity to tackle the symptoms and administer pain relief [23]. In addition, the fact that the general practitioner is heavily involved may play a role, as general practitioners in the Netherlands will often have known the patient and their family for many years.

### Methodological considerations

This study is based on a unique dataset that has been gathered within various care settings, in which the loved ones of patients with a wide variety of diseases have been included. One possible limitation of the dataset is whether it is representative of the Dutch population of patients who received palliative care. This was difficult to establish as there is no central register of such patients and the participating healthcare providers were all involved in the Palliative Care Improvement Programme, which may not be a random or representative selection. However, as many of our results are in line with previous research and/or common expectations, it is unlikely that potential issues with representativeness have greatly affected our results. An issue that may be more pressing with regard to the generalisability of results is whether our findings may also apply to palliative care in other countries. This is an issue in particular for the more extensive analysis that we were able to undertake, which cannot be compared to existing literature. Replication of this analysis in other countries would be welcomed as it is not possible to rule out that the way palliative care is organised in the Netherlands may have an effect.

With regard to the CQ-index questionnaire, it is important to mention that this is a validated questionnaire that looks not only at the general ratings for care but also at various aspects of palliative care. This involves asking the relatives concrete questions focusing on experiences (observations), rather than satisfaction (evaluations). The perceptions are partly about the care and support provided to the relatives themselves and partly about the care provided for the patient. There are no direct questions about the perspectives of the patients themselves, which is inherent in the fact that people cannot be asked questions about the care associated with their own deaths. Using the relatives as a proxy for the patients has been shown in earlier research to be a reliable way of reporting on the quality of care at the end of life [24].

## Conclusion

The quality of palliative care from the relatives’ perspective is highest when the patient died at home or in a hospice. The experiences are less positive in particular for patients who died in hospital or in residential elderly care. This applies both to the general ratings given to care for the patient and their loved ones and to specific aspects of palliative care such as care for the psychosocial well-being of the patient and their loved ones, the attitudes to them, autonomy, information in the final week before death, and expertise. This can partly be ascribed to differences in the patient population, but it also seems partly to be genuinely related to the care setting itself. This is an argument for letting people die at home, if they so wish and where feasible, as far as possible.

## Abbreviations

AIDS: Acquired Immunodeficiency Syndrome
COPD: Chronic Obstructive Pulmonary Disease
CQ-Index/CQI: Consumer Quality Index
CVA: Cerebrovascular accident
HIV: Human Immunodeficiency Virus
